# Effectiveness of aminophylline prophylaxis of renal impairment after coronary angiography in patients with chronic renal insufficiency

**DOI:** 10.4103/0971-4065.65300

**Published:** 2010-04

**Authors:** A. Rohani

**Affiliations:** Iran-Yasooj-Sajjad Hospital, Internal Medicine Ward, YASOOJ, Iran

**Keywords:** Aminophylline, coronary angioplasty, contrast nephropathy

## Abstract

This study was done to investigate whether aminophylline reduces the incidence of contrast induced nephropathy (CIN) after coronary angioplasty. Sixty patients who had serum creatinine concentration of > 1.3 mg/dl randomly received 250 mg IV aminophylline or placebo 30 minutes before coronary angiography. Serum creatinine and blood urea nitrogen were determined immediately before (base line) and at 24 and 48 hours after administration of contrast medium. The primary end point was the incidence of CIN. The incidence of CIN was 20% in placebo group and 13.3% in aminophylline group; older age was significantly associated with CIN: In this study, we could not demonstrate the prophylactic effect of a single infusion of 250 mg aminophylline, 30 minutes before administration of contrast media. A larger trial that incorporates the evaluation of clinically relevant outcomes is required to more adequately assess the role for aminophylline in CIN prevention.

## Introduction

The use of iodinated contrast medium during radiological procedure can result in nephropathy.[1] The intravascular administration of iodinated contrast agents is part of many diagnostic and therapeutic radiological procedures. Since these agents have no therapeutic value, its safety is important. The contrast agents that have been in use for many years have a high osmolarity and are ionic. Their administration has been associated with anaphylactic events, cardiovascular instability, and nephrotoxicity. Recently, low-osmolarity contrast agents, ionic and nonionic, have been introduced to reduce the incidence of side effects. Nephrotoxicity caused by contrast agents is usually identified according to Barrett’s definition (increase of serum creatinine of >0.5 mg/dl within 48 hours after contrast medium). In many studies, the condition is defined as an increase in the serum creatinine concentration of more than 25% or of more than 0.5 mg per deciliter within 48 hours after the administration of contrast agent.[2,3]

Contrast-induced nephrotoxicity (CIN) is rare in people with normal renal function. The most important risk factor is preexisting renal dysfunction, particularly that caused by diabetic nephropathy. Contrast media can lead to acute kidney injury, which results in longer hospitalization and increased mortality. Adenosine is a crucial mediator of contrast-induced nephropathy and functions further upstream than oxygen-free radicals (OFRs) and antioxidants.[4,5] There is evidence demonstrating that an elevated endogenous adenosine level may contribute to the pathophysiological process of acute reductions in kidney function following radiocontrast media exposure. Increased urinary excretion of adenosine has been demonstrated following the intravascular administration of radiocontrast media. Adenosine can induce sustained renal vasoconstriction and a reduction in glomerular filtration rate.

Adenosine receptor antagonists may attenuate the vasoconstrictive effects observed with radiocontrast media and preserve both renal blood flow and glomerular filtration perfusion pressure.[15–16] Therefore, it was the purpose of our study to investigate whether the adenosine antagonist aminophylline reduced the incidence of CIN after coronary angioplasty. We also characterized risk factors for CIN after coronary angioplasty.[6–10] The frequency of CIN strongly depends on a number of risk factors. In the worst case, CIN occurs in >50% of patients CIN, results in longer hospitalization and increased mortality. The in-hospital mortality of patients with CIN requiring dialysis can be as high as 36%>11, 12, 13. A large number of prophylactic procedures have been investigated. Data in patients who underwent coronary angiography are contradictory.

## Materials and Methods

Our institutional ethics review board has approved this study. Informed content was obtained from all patients. This is a double blinded trial. A total of 60 patients with stable serum creatinine of >1.3 mg/dl were prospectively randomized to receive either placebo (saline 0.9%; *n*=30) or 250 mg aminophylline (*n*=30). Placebo were applied intravenously as a short infusion (100 ml saline, 0.9%) 30 minutes before coronary angioplasty with >100 ml of the low osmolarity contrast medium (omnipaque). The stability of serum creatinine was verified by the comparison of baseline values immediately before contrast medium with >1 “screening” value of the preceding two days. Patients with a difference of >0.3 mg/dl were excluded.

Further exclusion criteria included pregnancy or contraindications to aminophylline (history of seizures, arrhythmia resulting in circulatory instability). Additional medications, including diuretics or angiotensin converting enzyme inhibitors (ACE-I), acetylsalicylic acid were not restricted in two groups and there are two patients in the aminophylline group and three in the placebo group who take ACE-I; only one patient received diuretics in placebo group. A fluid supply of 2 L/day was advised in both the groups. All patients received adequate intravenous volume expansion with isotonic crystalloid 1.0-1.5 ml/kg per hr for 3-12 hours before the procedure and for 6-24 hours afterward. Additional hydration was performed according to clinical examination, X-ray, and central venous pressures, if available.

### Evaluation criteria

Serum creatinine and blood urea nitrogen were determined, immediately before (baseline), and at, 24 and 48 hours after administration of contrast medium. The primary end point was the incidence of CIN.

### Statistical analysis

Sample size (*n*=60) was estimated assuming a CIN incidence of 13% in the aminophylline group and of 20% in the placebo group.

The predictive value of risk factors was evaluated by (1) comparison of the incidence (dichotomous parameters) and mean ± SD (continuous parameters) among patients with and without CIN; and multiple regression analysis (backward selection) with Y = maximum increase of serum creatinine compared with baseline Within 48 hours; the continuous variables of age, weight, creatinine, blood urea nitrogen, and amount of contrast medium; a dichotomous (yes/no) parameters of Aminophylline, diabetes, hypertension, nephrotoxic medication, proteinuria, impaired cardiac function.

## Results

### Demographic data and risk factors of the patients:

All patients were Caucasian and a mean age of 61.84+11.9 years. The most frequent reasons for renal impairment were hypertension 19.6% and diabetic nephropathy 17.4% and 11 patients had chronic renal failure of unknown origin. Patients receiving aminophylline and the controls were comparable with regard to risk factors for CIN, such as screening creatinine: 1.93±0.21 vs 1.84±0.54 mg/dl, *P*=0.41), baseline blood urea nitrogen (29.83±14.4 vs 33.3±11.2 mg/dl, *P*=0.46), amount of contrast media (200±89 vs 210±90 ml), prevalence of diabetes (21% vs 14.8% *P*=0.7). Mean baseline creatinine and screening creatinine levels were not significantly different for all patients (1.93±0.27 vs 1.59±0.54 mg/dl) or for the subgroups who received placebo (*P*=0.7) or aminophylline (*P*=0.8). Complete proteinuria diagnostics were performed in 18 patients (eight on aminophylline 10 on placebo). Overall, the time course was not different within the two subgroups. There was no adverse event during angioplasty.

The incidence of CIN, according to Barrett’s definition, was reduced from six patients (20%) who received placebo to 4 patients (13.3%) who received aminophylline prophylaxis (*P*=0.7) [Table 1]. Only two of the 10 patients with CIN were discharged with a serum creatinine as low as or lower than before receiving the contrast medium. Compared with baseline, mean serum creatinine of the 10 patients with CIN was significantly elevated at discharge (2.44±1.1 vs. 2.13±1.3 mg/dl *P*=0.0207). Therefore, contrast-induced renal impairment for a prolonged period must be assumed.

**Table 1:** Comparison of CR in both groups

	Baseline	24 hours	48 hours
Aminophylline	1.93±0.21	1.84±0.52	1.89±0.74
Placebo	1.84±0.54	1.89±0.74	2.06±1

Compared with baseline (1.93±0.21 mg/dl), mean creatinine reduced after using aminophylline at 24 hours (1.84±0.52 mg/dl), and 48 hours after administration of contrast medium (1.89±0.74 mg/dl). In placebo group, baseline serum creatinine compared (1.84±0.54 mg/dl) rose to 1.89±0.74 mg/dl at 24 hours and to 2.06±1 mg/dl, (*P*=0.99) 48 hours after receiving contrast medium.

### Evaluation of predictive parameters

The patients with CIN received significantly higher amounts of contrast medium (338+233 vs. 236+86 ml, *P*=0.0474). There were no sex difference (20% vs. 19% *P*=0.7). There was no difference seen in patients who received CCB before the procedure (11.1% vs. 9.2%, *P*=0.3); EF <30% or EF 30-50% (30.4% vs. 52.3% *P*=0.176) and in patients who have proteinuria or not (31.6% vs. 33% *P*=0.14). The risk factor in this study was only age >60 y (*P*=0.05).

## Discussion

In our study, we could not demonstrate the prophylactic effect of a single infusion of 250 mg aminophylline, 30 minutes before administration of contrast medium. The nephro-protective effect of aminophylline was thought mainly to be due to its glomerular adenosine antagonism. Contrast media osmotically irritate tubule cells. This results in an increased adenosine triphosphate turnover and the subsequent release of adenosine. In contrast to other tissues in which adenosine results in hyperemia, in the kidney, adenosine induces marked vasoconstriction of the vasa afferents via the adenosine - 1 - receptor. With an increase in renal impairment, the adenosine mediated vasoconstriction also increases. The importance of adenosine among other mediators of CIN, such as OFRs, prostaglandins, and angiotensin converting enzymes is further emphasized by the finding that the inhibition of adenosine re-uptake by dipyridamole significantly increases renal impairment by contrast media and leads to a 70% depression of total renal perfusion. Our 250 mg dose of aminophylline is greater that the minimum dose required blocking renal vascular adenosine receptors and would not be expected to yield a serum concentration above 100 *µ*gr/ml in most patients. Therefore, we did not administer the dosage using a weight-based strategy.

In previous studies, aminophylline was effective in dosages of 2.1 mg/kg IV, 2.6 mg/kg IV and 2.88 mg/kg once daily regardless of osmolality. Nevertheless, considering the weight range of the patients included in our study (50 to 100 kg), a weight-based dosing schedule should be applied in future studies. The plasma half life of aminophylline is about seven hours, which is longer than the half-life of contrast media in patients with normal or modestly impaired renal function. However, in patients with severely impaired renal function, the half-life of contrast medium might be longer than that of aminophylline. In these patients, a second dose of once daily or IV aminophylline might be beneficial.

However, patients included in our study are too small in number to compare the prophylactic efficacy of aminophylline. Whether a prophylaxis with acetylcysteine or a combination with aminophylline is superior to aminophylline alone, should be the subject of further studies.
